# DNA microarray profile of genes regulated by the epigenetic modifier sodium butyrate combined with etoposide in Burkitt’s lymphoma cells

**DOI:** 10.1186/1753-6561-7-S2-P5

**Published:** 2013-04-04

**Authors:** Ana C Ferreira, Patricia Severino, Claudete E Klumb

**Affiliations:** 1 Programa de Pesquisa em Hemato-Oncologia Molecular, Coordenação Geral Técnico-Científica, Instituto Nacional de Câncer-INCA, Rio de Janeiro, Brazil; 2 Centro de Pesquisa Experimental do Instituto Israelita de Ensino e Pesquisa Albert Einstein – IIEPAE, São Paulo, Brazil; 3 Departamento de Estomatologia da Faculdade de Odontologia, Universidade de São Paulo – USP, São Paulo, Brazil

## Background

The mechanisms underlying Burkitt’s Lymphoma (BL) chemoresistance and how it can be circumvented remain undetermined.The histone deacetylase inhibitors (HDACi) represent a novel class of agents which have demonstrated potent antitumor activity in preclinical models and promising clinical efficacy in cancer patients. The aim of this study was to evaluate the cell death enhancement effect of Sodium Butyrate (NaB), a HDACi combined with suboptimal concentration of etoposide (VP-16) and identify genes differentially regulated by this combination to provide rationale for novel drug combination patterns.

## Materials and methods

Raji BL cell line was treated with NaB isolated or combined with VP-16. Growth inhibition and cell cycle were analyzed in response to treatment using trypan blue exclusion assay and flow cytometry. Gene expression profiles were determined using One-Color Microarray-Based Gene Expression (Agilent Technologies) and analyzed on Feature Extraction v 9.5.1 software.

## Results

NaB/VP-16 combined treatment decreased cell proliferation and survival, and blocked cell cycle progression at G2/M with a concurrent decrease in S phase in Raji cells at 24h. Microarray profile showed upregulation of genes related to apoptosis, cell cycle arrest and response to DNA breaks. We also observed downregulation of genes related to cell cycle progression and angiogenesis.

## Conclusions

Alterations in critical genes involved in cell survival, angiogenesis, cell cycle and DNA damage response were identified. Pathways identified may represent potential targets for combined therapy protocols which have been emerging to improve treatment strategies to circumvent responseless BL patients.

## Financial support

APQ1/FAPERJ E26/111.330/2011; INCT Cancer, CNPq 573806/2008-0; FAPERJ E26/170.026/2008; PPSUS-2010.

